# Histidine-Rich Glycoprotein Can Prevent Development of Mouse Experimental Glioblastoma

**DOI:** 10.1371/journal.pone.0008536

**Published:** 2009-12-31

**Authors:** Maria Kärrlander, Nanna Lindberg, Tommie Olofsson, Marianne Kastemar, Anna-Karin Olsson, Lene Uhrbom

**Affiliations:** 1 Department of Genetics and Pathology, Uppsala University, Uppsala, Sweden; 2 The National Board of Forensic Medicine, Department of Forensic Medicine, Uppsala, Sweden; 3 Department of Medical Biochemistry and Microbiology, Uppsala University, Uppsala, Sweden; Institute of Cancer Research, United Kingdom

## Abstract

Extensive angiogenesis, formation of new capillaries from pre-existing blood vessels, is an important feature of malignant glioma. Several antiangiogenic drugs targeting vascular endothelial growth factor (VEGF) or its receptors are currently in clinical trials as therapy for high-grade glioma and bevacizumab was recently approved by the FDA for treatment of recurrent glioblastoma. However, the modest efficacy of these drugs and emerging problems with anti-VEGF treatment resistance welcome the development of alternative antiangiogenic therapies. One potential candidate is histidine-rich glycoprotein (HRG), a plasma protein with antiangiogenic properties that can inhibit endothelial cell adhesion and migration. We have used the RCAS/TV-A mouse model for gliomas to investigate the effect of HRG on brain tumor development. Tumors were induced with platelet-derived growth factor-B (PDGF-B), in the presence or absence of HRG. We found that HRG had little effect on tumor incidence but could significantly inhibit the development of malignant glioma and completely prevent the occurrence of grade IV tumors (glioblastoma).

## Introduction

Glioma is the most common and lethal type of primary brain tumor affecting adults. Gliomas are graded between I-IV following guidelines from the World Health Organization (WHO) [1]. Grade I-II tumors are considered benign and grade III-IV tumors malignant. The prognosis of patients diagnosed with malignant glioma is extremely poor with a median survival of 15 months for grade IV tumors [2]. Malignant glioma, in particular grade IV tumors (glioblastoma), often display ample microvascular proliferations which consists of multilayered, proliferating endothelial cells.

Formation of new capillaries is controlled by proangiogenic and antiangiogenic factors. One important proangiogenic factor is VEGF. In high-grade glioma VEGF-A in particular is upregulated in tumor cells [3], [4]. Also the receptors for VEGF-A, VEGFR1 and VEGFR2 expressed on endothelial cells, are upregulated in glioma [3], [5]. VEGF receptor signaling can promote angiogenesis, blood-vessel permeability, proliferation and migration of endothelial cells [6]. There are several anti-VEGF drugs that cause inhibition of VEGFR signaling including blocking antibodies and small-molecule tyrosine kinase inhibitors [7]. A humanized antibody against VEGF, bevacizumab (Avastin), was recently approved by the FDA for treatment of recurrent glioblastoma [8]. The decision was mainly based on two phase II trials where Bevacizumab caused a relatively high radiographic response rate and prolonged progression-free survival of the patients compared to historical controls [9], [10]. However, the effect on overall survival remains to be determined and in the end tumor progression inevitably resumed. In part, this may be due to the development of therapy resistance for which several different mechanisms are being proposed including increased invasiveness and co-option of cerebral vasculature [11] and upregulation of other proangiogenic pathways [12].

Histidine-rich glycoprotein (HRG) is a plasma protein of 75 kDa synthesized by liver parenchymal cells [13]. It is a multidomain protein displaying two cystatin-like regions at the N-terminus and a histidine-rich region (HRR) flanked by proline-rich regions (PRR) closer to the C-terminus [14], [15]. HRG can bind a variety of ligands such as haem, divalent cations, tropomyosin, plasminogen, plasmin, fibrinogen, thrombospondin, IgG, FcγR, C1q, heparin and heparan sulfate and has been shown to be involved in coagulation, immune response and angiogenesis [13], [16], [17], [18], [19].

HRG may both enhance and inhibit angiogenesis. HRG has been shown to act as a positive modulator of angiogenesis by inhibiting the antiangiogenic effect of TSP1 [20]. In contrast, several studies have suggested an antiangiogenic effect of HRG mediated by the HRR. HRG could inhibit endothelial cell tube formation and proliferation, block angiogenesis and induce apoptosis in endothelial cells [21]. Further, systemic administration of HRG was shown to be a potent inhibitor of tumor vascularization *in vivo*. In mice carrying syngeneic subcutaneous fibrosarcomas HRG treatment could significantly inhibit tumor growth and reduce tumor vascularization [22]. Also, when using the minimal active antiangiogenic domain of HRG (HRGP330, a 35 amino acid peptide) to treat subcutaneous human pancreatic adenocarcinomas in SCID mice tumor vascularization was significantly reduced [23]. The antiangiogenic effect of HRG is suggested to be due to disruption of focal adhesion function and hence reduced migration and adhesion of endothelial cells [22], [23], [24], [25].

The limited response of high-grade glioma patients to the current anti-VEGF drugs in combination with the increased risk of developing anti-VEGF resistance make additional antiangiogenic strategies welcome. In the present study we have used the RCAS/TV-A mouse model of glioma to study the effect of HRG on tumor formation. RCAS/TV-A is an orthotopic glioma model where induced tumors show many similarities with human glioma with regard to histopathology and genetics. The RCAS/TV-A model utilizes the selective ability of the avian retrovirus RCAS to infect cells which express the tv-a receptor. In *Ntv-a* mice, tv-a expression is driven by the nestin promoter causing infection to be restricted to neural/glial stem cells [26]. We used the *Ntv-a Arf-/-* mice and induced tumors with RCAS-PDGF-B, a combination that mainly produces high-grade gliomas [27] and analyzed how simultaneous expression of HRG would affect brain tumor development.

## Materials and Methods

### Ethics Statement

All animal experiments were done in accordance with the local animal ethics committee.

### Cloning of HRG into RCAS

We received the HRG cDNA (kind gift from Lena Claesson-Welsh) inserted in the pCEP-Pu2 vector [22]. A fragment containing the N-terminally His-tagged *HRG* cDNA in frame with BM40 signal sequence of pCEP-Pu2 [22], [28] was cloned into RCAS-Y (a derivative of RCASBP(A) [29]) via the pYAP6 adaptor plasmid. Correct orientation of the insert was confirmed by restriction enzyme mapping and sequencing with BigDye 3.1 (Applied Biosciences, Carlsbad, CA) according to the manufacturer's protocol and analyzed on an ABI PRISM 3700 DNA Analyzer (Applied Biosciences).

### Transfection of DF-1 Chicken Fibroblasts with RCAS-HRG

The DF-1 chicken fibroblast cell line was cultured as previously described [30]. The RCAS-*HRG* construct was transfected into the DF-1 cells using FuGene® 6 transfection agent (Roche Diagnostics, Bromma, Sweden) according to the manufacturer's protocol, cultured for at least two weeks to ensure efficient virus production and subsequent infection of the cells before they were used directly for injection or to harvest conditioned media.

### HRG Protein Purification

Conditioned media was collected from DF-1 RCAS-HRG cells after 3-5 days incubation. His-tagged HRG was purified from the media using Ni-NTA agarose (Qiagen, Germantown, MD). Eluted fractions were tested for protein content using the BCA protein detection kit (Pierce/Thermo Scientific, Rockford, IL). Positive fractions were pooled and dialyzed against PBS. HRG protein in PBS buffer was dialyzed to a buffer containing TBS/1 mM ZnCl_2_, since Zn^2+^ is required for its antiangiogenic activity [24].

### Chemotaxis Assay

Human umbilical vein endothelial cells (HUVEC) were cultured in complete Endothelial Cell Basal Medium MV2 (Promo Cell, Heidelberg, Germany). The chemotaxis assay was performed using a modified Boyden chamber with 8 µm micropore polycarbonate filters (PFB8-50; Neuro Probe Inc., Gaithersburg, MD) coated with type-1 collagen solution at 100 µg/ml (Vitrogen 100; Collagen Corp, Palo Alto, CA). HUVEC that had been starved over night in 0.5% FCS/MV2 without additions were trypsinized and resuspended at 4×10^5^ cells/ml in MV2 without additions, 10 µM ZnCl_2_, 0.25% BSA and trasylol at 1000 KIE/ml, to stop trypsin activity. The cell suspension was added in the upper chamber and growth factor (VEGF-A at 10 ng/ml; Peprotech, Rocky Hill, NJ) in the lower chamber. HRG was added both in the upper and in the lower chamber at 100 ng/ml. After 5 h at 37°C cells that had migrated through the filter were stained with Giemsa and counted. Data is presented in relation to the number of cells that migrated through the filter in the absence of VEGF-A or HRG. Statistical analysis was done with the unpaired t-test using GraphPad Prism Software 4.0 analysis program.

### *In vitro* Infection of Glial Stem Cells and Proliferation Assay

Primary glial stem cell cultures were established from neonatal *Ntv-a Arf-/-* mice and cultured in N2 medium on polyornithine/fibronectin coated tissue culture plates with addition of 10 ng/ml FGF-2, as previously described [30]. Cells were infected for five days with sterile filtered conditioned N2 media from DF-1 cells producing RCAS-*HRG*, RCAS-*PDGFB-HA* [31] or RCAS-*eGFP*. After infection equal number of cells were seeded in 35-mm dishes for proliferation assay, and on coverslips for immunofluorescence staining. To determine proliferation cells were counted at day 1, 3 and 7. Statistical analysis was done with the unpaired t-test using GraphPad Prism Software 4.0 analysis program. Immunofluorescence staining was done as described [30] with primary antibodies rabbit anti-HRG (0119) [22], rabbit anti-eGFP (Abcam, Cambridge, UK) or rabbit anti-HA (Santa Cruz, Santa Cruz, CA). After incubation with secondary antibody anti-rabbit Alexa555 (Invitrogen, Carlsbad, CA) coverslips were mounted in DAPI containing ImmuMount (Shandon, Pittsburgh, PA) and pictures taken using a Zeiss 510 Meta confocal microscope.

### Western Blot Analysis

Conditioned media was collected from virus producing cells and infected glial stem cells and mixed with 4x loading buffer (Invitrogen) and reducing agent (Invitrogen) before heated at 95°C for 5 min. Western blot analysis was done as described [32]. A primary antibody 0119 [22] raised against the His/Pro-region was diluted 1∶2000 in 1% non-fat dry milk (Bio-Rad, Hercules, CA) in PBS-T and incubated at 4°C overnight. PDGF-B was detected using an antibody against HA (1∶800, Nordic Biosite, Täby, Sweden). Purified recombinant HRG was used as positive control, medium from DF-1 cells transfected/infected with RCAS-X (empty RCAS vector) as negative control.

### *In vivo* Glioma Studies

Neonatal mice were intracerebrally injected with 5×10^5^ RCAS virus producing DF-1 chicken fibroblasts (RCAS-*PDGFB-ires-eGFP* [33] co-injected with either RCAS-*HRG* or RCAS-X). Mice were monitored three times per week and killed when ill or at twelve weeks. The brains were either fixed in 4% formalin for at least 48 h and embedded in paraffin or fixed in 4% PFA for 1 hour at 4°C, immersed in 30% sucrose over night and embedded in OCT Compound (Sakura Finetech, Torrance, CA). Brain tissue was sectioned, hematoxylin and eosin stained (H&E) and analyzed in a blinded procedure by a neuropathologist (TO) for presence and grade of tumors. Statistical analysis of tumor incidence and grade distribution with Fisher's exact test and Kaplan-Meier analysis of overall survival was done using GraphPad Prism Software 4.0 analysis program.

### Immunohistochemical Analysis of Tumors

Immunohistochemical staining was done as described [30]. Before applying the CD31 antibody (goat, 1∶200, Santa Cruz) slides were blocked in 20% normal rabbit serum. Pictures were taken using a Leica bright field microscope. Immunohistochemical analysis was performed on 13 out of 13 PDGF-B+HRG (P+H) tumors and 13 out of 22 PDGF-B+RCAS-X (P+X tumors).

### DNA Extraction and Detection of Virally Inserted Genes

DNA was extracted from paraffin embedded tissues, ten 10 µm tissue sections per sample. After deparaffinisation in xylene and rehydration in ethanol sections were scraped off the glass and digested in 50 mM Tris-HCl pH 8.0, 100 mM EDTA pH 8.0, 100 mM NaCl and 1% SDS with addition of 0.25 mg/ml Proteinase K (Roche) in 55°C for 2 hours. Proteins were removed using Protein precipitation solution (Promega, Madison, WI) and DNA precipitated with isopropanol, washed with ethanol and reconstituted in 10 mM Tris-HCL pH 8.0. For PCR detection 100 ng DNA was used with PuReTaq Ready-To-Go™ PCR Beads (GE Healthcare, Uppsala, Sweden) system. Primers used specific for human PDGF-B were forward primer B1-F (5′-TGCTGCTACCTGCGTCTGGTC) and reverse primer R1 (5′-ATGTTCAGGTCCAACTCGGC) yielding a 158 bp fragment. The human HRG primers used were forward F2 (5′- TTAAGAAGGCHAGGCCCAGGTAAA) and reverse R2 (5′-TTTAGAGGATGTTTGTGGTGCGGC) yielding a 158 bp fragment. PCR setup used was 95°C for 5 minutes followed by either 35 (PDGFB) or 40 (HRG) cycles of 95°C for 30 s, 55°C for 30 s and 72°C for 1 minute ending at 72°C for 10 minutes. PCR products were visualized on agarose gels and pictures taken using Gel Foto System 1000 (Techtum Lab, Umeå, Sweden).

### RNA Extraction and Detection of RCAS Mediated Gene Expression

Total RNA was extracted from frozen brain tumor tissue, eight slides of 20 µm tissue sections per sample using TRIzol® reagent (Invitrogen). cDNA was made through reverse transcription of 1 µg of total RNA using random primers (S12545, New England Biolabs, Ipswich, MA) and M-MuLV reverse transcriptase (New England Biolabs) and subsequently 100 ng of cDNA was used for PCR detection of PDGF-B and HRG of human origin used in the RCAS vectors utilizing the PuReTaq Ready-To-Go™ PCR Beads (GE Healthcare, Uppsala, Sweden) system. RNA from U-343MG-a was used as positive control for PDGF-B, DF-1 RCAS-*HRG* as positive control for HRG and frozen brain tissue from an FVB/N mouse as negative control. Primers were the same as for DNA detection. PCR setup was 95°C for 5 minutes followed by either 30 (PDGFB) or 40 (HRG) cycles of 95°C for 30 s, 55°C for 30 s and 72°C for 1 minute ending at 72°C for 10 minutes. PCR products were visualized on agarose gels and pictures taken.

## Results

### DF-1 Cells Infected with RCAS-HRG Produced Functional HRG

**Figure 1:**
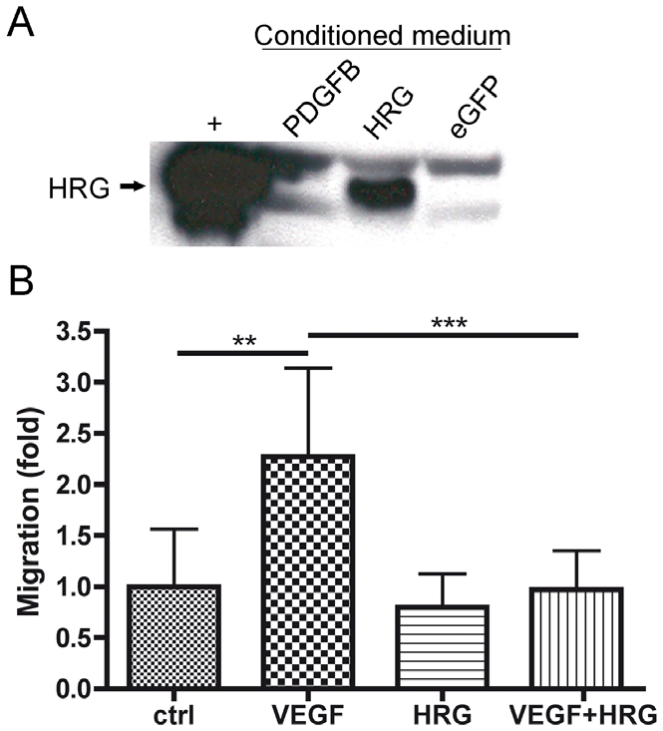
RCAS-HRG infected DF-1 cells could produce functional HRG. (**A**) Viral produced HRG protein could be detected by western blot in conditioned media from DF-1 cells infected with RCAS-HRG but not from RCAS-PDGFB or RCAS-eGFP infected cells. Purified HRG was used as a positive control (+). (**B**) Viral produced HRG could significantly inhibit migration of HUVEC cells towards VEGF. t-test, ** p<0.01, *** p<0.001.

The HRG gene was cloned into the RCAS-Y vector and the construct transfected into DF-1 cells for virus production. Western blot of conditioned media from DF-1 RCAS-*HRG* cells showed that HRG protein was produced (Figure 1A). To assure that the virally produced HRG was functional a chemotaxis assay was performed. Human umbilical vein endothelial cells (HUVEC) were stimulated to migrate towards VEGF in a modified Boyden chamber in the presence or absence of purified HRG (Figure 1B). HRG on its own did not affect endothelial cell migration. As expected VEGF could induce a more than two-fold increase in migration compared to control but when VEGF and HRG were added together the level of migration was the same as that of control. Thus, the virally produced HRG was able to significantly inhibit VEGF induced endothelial cell migration (p = 0.0007), in line with previous data on HRG [22], [23], [24].

### HRG Did Not Affect Primary Glial Cell Proliferation

**Figure 2:**
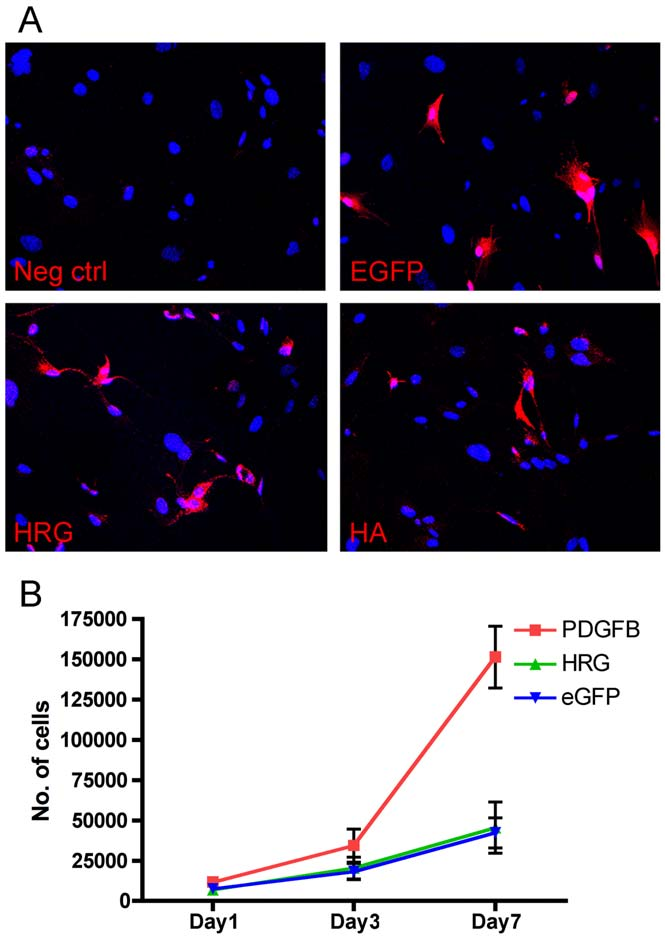
HRG had no effect on primary glial cell proliferation. (**A**) Primary glial cells were infected with RCAS-eGFP, RCAS-HRG or RCAS-PDGFB-HA and expression of the viral transduced proteins was analyzed with immunocytochemistry. The infection efficiency was similar in all conditions. (**B**) Proliferation assay on infected cells showed no effect of HRG compared to control cells on glial cell proliferation at day 7. Curves show the mean (±SEM) from three independent experiments for HRG and eGFP, and two independent experiments for PDGF-B.

To analyze if HRG had a direct effect on glial cell proliferation, primary glial stem cell cultures were set up from neonatal *Ntv-a Arf-/-* mouse brains. The cells were infected with RCAS viruses expressing HRG, PDGFB-HA [31] or eGFP (control) and subsequently stained for GFP, HRG and HA to determine infection efficiency (Figure 2A). For proliferation analysis equal number of cells were seeded on day 0 and cell numbers determined day 1, 3 and 7. As shown previously for primary glial cells [27], [30] PDGF-B could stimulate glial stem cell proliferation compared to control infected cells (p = 0.0101), while HRG had no effect on proliferation compared to control cells (p = 0.8683) (Figure 2B).

### HRG Could Inhibit PDGF-B Induced Development of Grade IV Glioma

To analyze the effect of HRG on glioma development neonatal *Ntv-a Arf-/-* mice were intracranially injected with equal numbers of DF-1 RCAS-*PDGF-B-eGFP* [33] and DF-1 RCAS-*HRG* or DF-1 RCAS-X (control virus, complete RCAS without inserted exogenous gene). Mice were monitored for 12 weeks. There was a small but non-significant effect on tumor incidence (Fischer's exact test, p = 0.514) by HRG with 42% in PDGF-B+X (P+X) and 34% in PDGF-B+HRG (P+H) injected mice (Table 1).

**Table 1:** Tumor incidence in *Ntv-a Arf*-/- mice injected with PDGF-B+X (P+X) or PDGF-B+HRG (P+H) RCAS viruses.

RCAS	Injected	No of tumors	Incidence
P+X	52	22	42%
P+H	38	13	34%

**Figure 3:**
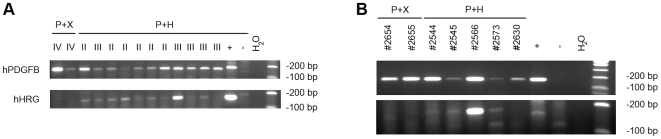
Presence and expression of viral transduced PDGF-B and HRG in tumors. (**A**) Insertion of the viral transduced human PDGF-B and HRG cDNA in genomic DNA prepared from PDGF-B+X (P+X) and PDGF-B+HRG (P+H) induced tumors. The tumor grade is given above each sample. Genomic DNA from U-706MG-a cells was used as positive control (+), and genomic DNA from an untreated mouse was used as negative control (−). (**B**) Expression of human PDGF-B and HRG mRNA in P+X and P+H tumors. RNA extracted from U-343MG and DF-1 RCAS-HRG cells were used as positive control for PDGF-B and HRG, respectively (+), and RNA from an untreated mouse brain was used as negative control (−).

To determine HRG expression in tumors we initially tried immunostainings for HRG protein on tumor sections. Unfortunately, the HRG antibody available to us did not work on formalin-fixed, paraffin-embedded tissue sections despite extensive modifications of the protocol. Instead, since RCAS is a retrovirus we decided to analyze RCAS mediated insertion of PDGF-B and HRG cDNA in genomic DNA isolated from tumor tissue. DNA was prepared from formalin-fixed paraffin embedded tissue sections of two P+X and eleven P+H injected, tumor-bearing mice. Two of the P+H tumors could not be analyzed since these tumors were very small (both were grade II) and there was no tumor tissue remaining. PCR was performed with primers specific for human PDGF-B and human HRG cDNA. As expected, since PDGF-B is required for tumor development in this model, we found human PDGF-B cDNA in all genomic DNA samples analyzed (Figure 3A). We also found that all P+H tumors were positive for human HRG cDNA (Figure 3A). To analyze if the virally transduced PDGF-B and HRG genes were expressed we isolated RNA from a small set of tumors (two P+X and five P+H) that had been frozen prior to RNA preparation. Unfortunately, the fixation of these brains had failed and the histopathology could not be properly evaluated. Thus, these tumors were not included in Table 1 or the statistical evaluation of the effect of HRG below. Expression of human PDGF-B and HRG mRNA was detected with RT-PCR (Figure 3B). PDGF-B mRNA could be found in all samples verifying the presence of tumor tissue. HRG mRNA was clearly detected in one P+H sample (#2566) and weakly in three samples (#2544, #2545, #2573). One sample (#2630) failed to show a reliable signal. The fact that HRG expression could not be consistently detected in one of the P+H tumors and only weakly in some could be due to that HRG was expressed below the level of detection, diluted by mRNA from surrounding normal cells, since P+H tumors generally had both lower tumor cell density and were smaller in size. There is also the possibility that the tumors were polyclonal and therefore had expression of HRG in some parts but not in others, or even that occasional tumors could have been induced from cells solely infected with RCAS-PDGF-B.

**Figure 4:**
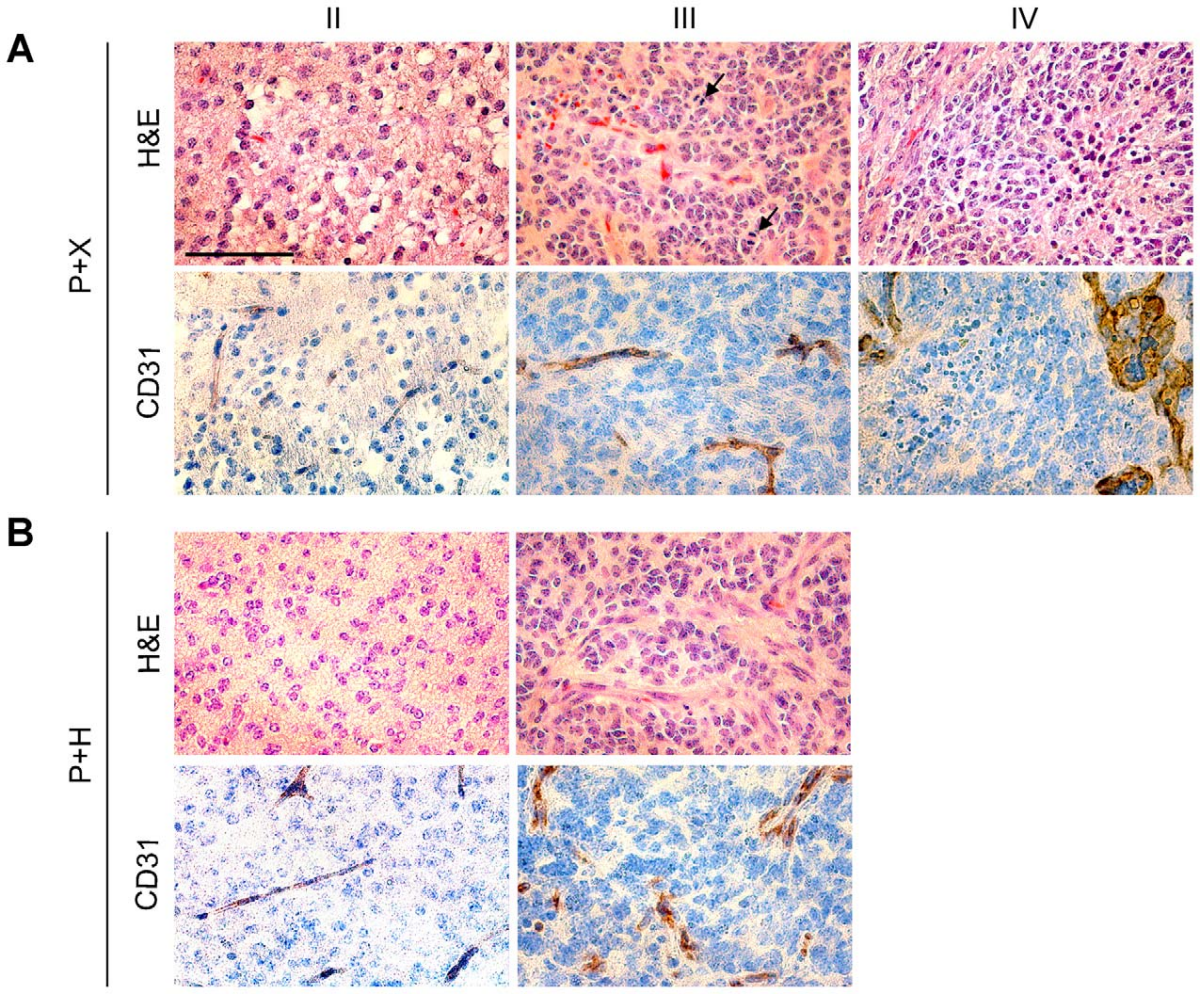
Tumor histopathology and vessel morphology. (**A**) Histopathology (H&E) of representative grade II–IV tumors induced with PDGF-B+X (P+X) and corresponding immunostainings for CD31. Arrows indicate mitoses. (**B**) Histopathology (H&E) of representative grade II–III PDGF-B+HRG (P+H) tumors and corresponding CD31 stainings. Bar = 100 µm.

**Figure 5:**
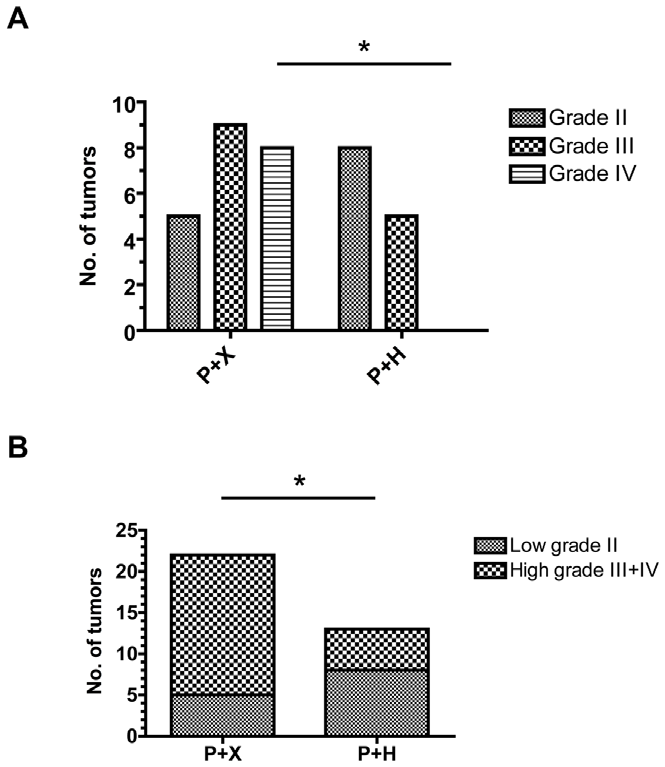
Distribution of tumor malignancy grades. (**A**) Distribution of tumor grades (II–IV) in PDGF-B+X (P+X) and PDGF-B+HRG (P+H) injected *Ntv-a Arf-/-* mice. * p<0,05 (**B**) Distribution of low grade (II) versus malignant (III+IV) glioma. *p<0,05.

Tumor histopathology was analyzed and tumors were graded from II-IV. Grade II tumors grew diffusely into the brain parenchyma and consisted of small, round tumor cells (Figure 4A–B, upper left panels). Grade III tumors displayed mitotic figures, cellular and/or nuclear pleomorphism, and increased microvascular density (Figure 4A–B, upper middle panels). To be classified as a grade IV tumor, corresponding to glioblastoma, the addition of pseudopalisading necrosis and/or microvascular proliferations consisting of multilayered proliferating endothelial cells had to be present (Figure 4A, right panel). In control mice (P+X) tumors of all grades were found with the majority of tumors being high-grade gliomas (grade III–IV, Figure 4A and 5). There was a clear shift towards lower grade tumors (grade II) in P+H injected mice (Fischer's exact test, p = 0.0328) and grade IV tumors were entirely absent in mice that had received HRG (P+H) (Fischer's exact test, p = 0.019, Figure 4B and 5).

### Reduced Aberrant Tumor Vascularization in HRG Injected Mice

The tumors were analyzed for expression of various proteins with immunohistochemical stainings. Tumors of all grades expressed the glial cell markers OLIG2, SOX2 and PDGFRα and there was no difference in staining pattern between P+X and P+H injected mice (data not shown). Immunostainings for Ki67 and quantification of positive cells showed no difference in tumors of the same grade between the two groups (data not shown) further supporting the *in vitro* data that HRG did not seem to have a direct effect on glial cell proliferation. To determine the extent of vascularization (presence of endothelial cells) in the tumors CD31 stainings were performed (Figure 4A–B, lower panels). Tumors of the same grade from both P+X and P+H injected mice were similarly positive with increased number of positive cells with malignancy grade. Normal brain (data not shown) and low-grade tumors displayed presence of thin and organized vessels whereas high-grade tumors contained vessels that often appeared disorganized and multi-layered, suggesting that an increase in microvascular proliferation is a prerequisite for malignant glioma to occur.

To conclude, our investigation shows that expression of HRG in tumor cells can significantly inhibit the development of high-grade glioma and the mechanism is suggested to be through inhibition of tumor angiogenesis.

## Discussion

To date, there are three antiangiogenic drugs targeting VEGF signaling (bevacizumab, sunitinib, sorafenib) approved for cancer therapy [34]. Bevacizumab was the first antiangiogenic drug approved for use in cancer patients. In combination with cytotoxic drugs bevacizumab is indicated in treatment of metastatic colorectal cancer [35], non-small cell lung cancer [36] and metastatic HER2-negative breast cancer [37], and recently as single treatment of recurrent glioblastoma [9], [10]. The beneficial effect of combining anti-VEGF treatment with cytotoxic agents is suggested to be primarily due to improved drug delivery to the tumor [38]. Large excess of VEGF can induce vascular permeability and increased interstitial fluid pressure in the tumor that interferes with efficient drug delivery. VEGF inhibition is believed to cause normalization of the vasculature that improves transport across the vessel wall. However, two recent studies [39], [40] report that mice treated with VEGF-inhibitors display increased tumor invasiveness and metastasis, although growth of the primary tumor was reduced. These findings may have important implication for how drugs targeting VEGF-receptor signaling are used in the clinic. It is therefore of high priority to find out whether this is a specific effect of VEGF-inhibitors or a common theme also for other antiangiogenic strategies.

One hallmark feature of malignant glioma is a marked increase in microvascular hyperplasias caused by hypoxia-induced VEGF signaling. These tumor blood vessels are perturbed and leaky resulting in increased interstitial fluid pressure, peritumoral edema and incomplete distribution of oxygen and other blood-delivered factors, including chemotherapeutic drugs, to the tumor [41]. In addition, it has recently been shown that tumor vessels can function as a vascular niche for maintainance of glioma cancer stem cells by producing an as yet unknown factor, and that reduction of blood vessels caused suppression of tumor growth and significantly decreased the number of self-renewing tumor cells [42]. Thus, by suppressing angiogenesis in glioma several tumor promoting mechanisms will be inhibited.

We have investigated the effect of the antiangiogenic protein HRG on orthotopic PDGF-B induced glioma development *in vivo* using the *Ntv-a Arf*-/- mice that generate a high proportion of malignant tumors. Previous studies had shown that it would be possible to reach therapeutic concentrations of HRG in mice by systemic administration [22], [23]. However, to avoid the potential problem of not getting HRG through the blood-brain barrier we decided to express the protein in the tumor cells in this initial study. We found that HRG could significantly inhibit the development of malignant tumors and prevent development of glioblastoma. When analysing the tumor blood vessels we found that high-grade tumors contained disorganised and often multi-layered vessels that could not be found in low-grade tumors or normal brain. This is an important finding, suggesting that administration of HRG or HRG-derived synthetic peptides could be potentially valuable in treatment of glioma patients, and perhaps even more so in combination with already established treatment modalities. One fatal characteristic of human glioma is the strong inherent propensity of even benign tumor cells to malignant progression. Our results imply that HRG therapy may be effective to prevent tumor progression of low-grade to high-grade glioma.

Survival is the most used parameter in clinical trials of glioma therapy and patients with low-grade glioma have significantly longer survival compared to patients with malignant glioma. There was no significant effect by HRG on survival of tumor-bearing mice (data not shown). We believe that the limited time for the *in vivo* studies (12 weeks) may not have been sufficient to detect such an effect since many tumors were found in asymptomatic mice at the termination of the experiment. The fact that HRG significantly could inhibit development of malignant glioma strongly suggests that it also could have a positive effect on survival in a more long-term study. Also, since HRG was administered as a separate virus together with the tumor inducing oncogene double infection of all target cells were required for efficient HRG expression in the tumors. In some mice the expression of HRG and PDGF-B could have occurred in separate cells leading to no or only regional expression of HRG in those tumors, which may be one reason for why HRG could not be detected in all P+H tumors.

The current antiangiogenic drugs are promising but not optimal and problems with VEGF-induced resistance to treatment are emerging. Therefore, it is vital to find alternative antiangiogenic treatment strategies not targeting VEGF. The fact that HRG as a single treatment regimen could inhibit development of malignant gliomas and completely suppress glioblastoma development is encouraging and stimulates further studies using HRG alone and in combination with cytotoxic drugs and receptor tyrosine kinase (RTK) inhibitors, including VEGF inhibitors, to treat experimental glioma of all grades.
